# Prevalence and determinants of unintended pregnancy among rural reproductive age women in Ethiopia

**DOI:** 10.1038/s41598-024-81067-w

**Published:** 2025-01-05

**Authors:** Melak Jejaw, Kaleb Assegid Demissie, Misganaw Guadie Tiruneh, Kaleab Mesfin Abera, Yawkal Tsega, Abel Endawkie, Wubshet D. Negash, Amare Mesfin Workie, Lamrot Yohannes, Mihret Getnet, Nigusu Worku, Adina Yeshambel Belay, Lakew Asmare, Hiwot Tadesse Alemu, Demiss Mulatu Geberu, Asebe Hagos

**Affiliations:** 1 Department of Health Systems and Policy, Institute of Public Health, College of Medicine and Health Sciences, University of Gondar, Gondar, Ethiopia; 2 Department of Health Policy and Systems, Institute of Public Health, College of Medicine and Health Sciences, Wollo University, Dessie, Ethiopia; 3 Department of Health System and Management, School of Public Health, College of Medicine and Health Sciences, Wollo University, Dessie, Ethiopia; 4 Department of Epidemiology and Biostatistics, School of Public Health, College of Medicine and Health Sciences, Wollo University, Dessie, Ethiopia; 5 National Center for Epidemiology and Population Health, the Australian National University, Canberra, Australia; 6 Department of Nutrition and Dietetics, Institute of Public Health, College of Medicine and Health Science, University of Gondar, Gondar, Ethiopia; 7 Department of Environmental and Occupational Health and Safety, Institute of Public Health, College of Medicine and Health Sciences, University of Gondar, Gondar, Ethiopia; 8 Department of Human Physiology, School of Medicine, College of Medicine and Health Sciences, University of Gondar, Gondar, Ethiopia; 9 Department of Epidemiology and Biostatistics, Institute of Public Health, College of Medicine and Health Sciences, University of Gondar, Gondar, Ethiopia

**Keywords:** Magnitude, Unintended pregnancy, Multilevel analysis, Ethiopia, Health care, Health occupations, Medical research

## Abstract

Despite growing utilization of family planning in Ethiopia, many pregnancies in rural areas are still unintended and unintended pregnancy remains a major global challenge in public and reproductive health, with devastating impact on women and child health, and the general public. Hence, this study was aimed to determine the prevalence and associated factors of unintended pregnancy in rural women of Ethiopia. This study used a 2016 Ethiopian Demography and Health Survey data. A total weighted samples of 974 reproductive-aged rural women were included in this analysis. Multilevel mixed logistic regression analysis was employed to consider the effect of hierarchal nature of EDHS data using Stata version 14 to determine individual and community level factors. Variables significantly associated with unintended pregnancy were declared at p-value < 0.05 with adjusted odds ratio and 95% confidence interval (CI). The prevalence of unintended pregnancy in rural women was 31.66% ( 95%CI 28.8%, 34.66%). Never had media exposure (AOR: 2.67, 95%CI 1.48, 4.83), don’t have work (AOR: 0.33, 95%CI 0.21, 0.52), being from household size of one to three (AOR: 0.44 95%CI 0.2, 0.96), being primiparous (AOR: 0.41, 95%CI 0.17, 0.99), women from poor families (AOR: 2.4, 95%CI 1.24, 4.56), lacking the intention to use contraceptive (AOR: 0.24, 95%CI 0.14, 0.44) were individual-level factors significantly associated with unintended pregnancy. Women from large central region (AOR: 4.2, 95%CI 1.19, 14.62) and being from poor community wealth status (AOR: 4.3, 95%CI 1.85, 10.22) were community-level factors statistically associated with unintended pregnancy. The present study prevalence of unintended pregnancy in rural women was relatively high. Maternal occupation, household size, media exposure, parity, women wealth, intention to use contraceptive, region and community level wealth were factors statistically associated with unintended pregnancy. Hence, demographer and public health practitioners has to give great emphasis on designing an intervention with implementation strategies to increase accessibility of media for reproductive-age women’s and improve women financial capacity, and strengthen maternal health services. These strategies helps to decrease adverse birth outcomes associated with unintended pregnancy in rural areas.

## Introduction

Unintended pregnancy refers to a pregnancy that happened when a woman either did not want to become pregnant at that time or did not have a plan to become a pregnant in the future^1,2^. Mothers with unintended pregnancy may experiencing premature delivery, and other adverse pregnancy associated outcomes^3,4^.

Unintended pregnancy remains a neglected global public health challenge. Globally, about 121 million unintended pregnancies occurred annually with on average of 331,000 unintended pregnancies per day from 2015 to 2019. The prevalence of unintended pregnancy in Sub-Saharan Africa was 29% with ranges from 10.8% in Nigeria to 54.5% in Namibia. Additionally, about 14 million unintended pregnancies occurs per year among sexually active women^5^.

Unintended pregnancies may occurs due to different reasons, including inability to use contraception, discontinuation of contraception methods, contraceptive failure, inappropriate or inconsistent use of contraception, and lack of knowledge regarding family planning^6^.

Unintended pregnancies remarkably increases in low and middle income countries, leading to considerable adverse consequences for both mother and child. These consequences includes decrease mother to child relationship quality, increase risk of physical abuse and violence against women, and social stigma because of cultural and religious factors, and negatively affected women-partner relationships within the communities^7–9^. Additionally, unintended pregnant mothers are high risk to develop serious complications, including unsafe abortion, increase crime rate, maternal depression, induces family and parent stress, decrease workforce productivity and limit job options, and decrease academic performance till derail from educational plan specially rural women. According to 2020 WHO report, approximately 60% of unintended pregnancies were ended with abortion and about 45% of the abortions were unsafe. The other serious complications of unintended pregnancies related abortions are hemorrhage, uterine perforation, cervical injury and infection due to procedure that may leads to maternal death. Moreover, approximately seven million unintended pregnancies induced unsafe abortion leads to hospital admission in each year in developing countries^10–13^. Furthermore, unintentionally pregnant mothers usually starts their antenatal care visit lately since they are unhappy and careless, and disinterested to give birth at the health facilities^4^. Generally, unintended pregnancies has negative impacts on maternal personal life and their families, impose financial and social costs, and substantially contributed to maternal morbidity and mortality^14^.

Prior study conducted in Sub Saharan Africa (SSA) countries have showed that unintended pregnancies are more common among women who lives in rural areas^5^. Besides, the 2016 Ethiopian DHS report showed unintended and teenage pregnancies are more common in rural areas^5^. Moreover, women living in rural areas are more often less educated, has limited access to family planning services due to distance problem to health facilities and limited decision making power, more likely to have parity, and financially unsecured^15^. Furthermore, mostly, women living in rural areas don’t have access to social media to obtain information about maternal and child health service and to make judgments related to different issue^16^.

Even though there is a growing utilization of family planning in Ethiopia, unintended pregnancies remain the main public health challenge, with prevalence rates ranging 13.7–41.5%^17–19^.

From various global and local reviewed literatures socio-demographic factors such as maternal age^6,18,20–22^, maternal educational level^22–24^, religion^19,25,26^, maternal marital status^27–30^, home distance from the nearest health facility^28^ parity^28,29^, household size^31,32^, income/wealth status^5,23,26^, knowledge of ovulation cycle^33^, knowledge of family planning^6^, ever had history of terminated pregnancy^18,21^, residence^6,26^ and region^22,31^ are the key factors statistically associated with unintended pregnancy.

The sustainable development goals (SDGs) has targets to ensure the right of all women and couple to choose the number and spacing their children with global ambition of all births are wanted and all child are valued by 2030^11^. Vigorous efforts are needed to alleviate unintended pregnancies which in turn prevents the adverse outcomes of abortion, save financial and social costs, reduce maternal and child mortality^34^.

Despite unintended pregnancies have aforementioned adverse outcomes, as to authors search, there is paucity of evidence about magnitude and determinants of unintended pregnancy among rural women based on nationally representative data in Ethiopia. Hence, this study aimed to determine the prevalence and associated factors of unintended pregnancy among rural women in Ethiopia. This study finding will provide knowledge to health workers and policy makers to understand the magnitude of unintended pregnancy and its associated factors to fill gaps by developing attainable intervention with implementation strategies.

## Methods

### Data source and population

This study used a nationally representative Ethiopian Demography and Health Survey (EDHS) data collected from nine region and two city administration from January 18, 2016, to June 27, 2016^36^.

The 2016 EDHS survey used a two stage stratified sampling technique to select a sample. First, regions were stratified in to urban and rural areas. Every stratum was clustered into 645 enumeration areas( 202 in urban and 443 in rural) by using the 2007 Ethiopian Population and Housing Census (PHC)^35^ as sampling frame. Each clustered were selected by using probability proportional size allocation. Secondly, 28 household per cluster with total 16,650 households (15,683 female respondents and 12,688 male respondents) were selected by using equal probability systematic sampling technique. The survey data was focus on reproductive health (fertility and fertility preference, marriage, awareness and the use of family planning methods), adult and childhood morbidity and mortality, and reproductive women awareness and attitudes towards HIV/AIDS and other important public health issues. The overall information about the sampling procedure and the questionnaire can be accessed from the EDHS 2016 report^36^. This study used the women’s data (IR data) set of 2016 EDHS, which was obtained from the demographic and health survey (DHS) program website (http://www.measuredhs.com). The sample data were weighted by using v005 (the individual weighted for women) to ensure the representativeness of the sample, to obtained reliable estimation and standard errors since the overall probability of selecting a household is not constant.

**Fig. 1:**
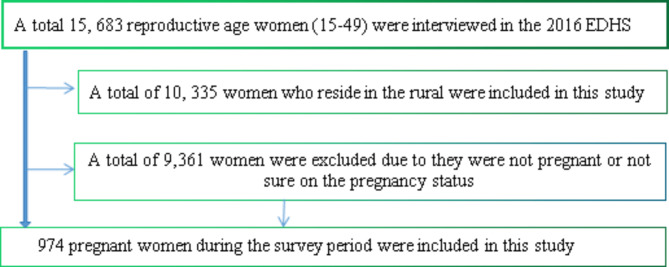
Diagram representation of selecting the sample from the EDHS 2016 data.

For this study analysis, a total weighted sample of 974 pregnant women were selected from the total of 10, 335 rural dwellers reproductive age women(15–49 years) interviewed in the 2016 EDHS (Fig. 1).

### Study variables and definition

The dependent variable name for this study was unintended pregnancy. The variable name in the EDHS datasets is “current pregnancy wanted”. The reproductive age women who participated in this survey were asked if they were currently pregnant. Those women who answered “yes” were further asked whether they wanted their current pregnancy or not. They had three options to answer the questions such as “wanted then”, “wanted later” and “not at all”. Pregnancies were considered as unintended if the pregnancy were either unwanted(the mother didn’t want to become pregnant at all) or the pregnancy was mistimed( the mother didn’t have plan to become pregnant at that time but wanted later)^13^. Then the dependent variable of unintended pregnancy was dichotomous created by re-coding the original variable as “wanted then”=0 as “intended”, “wanted later” and “not at all” = 1 as “unintended”.

The independent variables were individual level variables such as maternal age, maternal occupation status, maternal educational status, marital status, religion, parity, household size, wealth status, media exposure, ever had of termination pregnancy, knowledge of ovulation cycle, sex of household head, unmet need for contraceptive, fertility preference, knowledge of contraception, intention of contraceptive use, and community level variable such as region, community perceived distance from the health facility, community women educational status, community women wealth status^37^, community women media exposure, community women awareness of modern contraceptives. Some variables were recorded for simplicity of this study analysis such as wealth status of poorest and poorer category were re-coded as poor = 1, those above the poorest wealth category and below the high category were leveled as middle = 2, whereas richest and richer wealth category were re-coded as rich = 3. On the other hand, region was recorded similarly with the previous study in Ethiopia as “small peripheral region” that includes Afar, Somali, Benishangul, and Gambela, and the “large central region” which includes Tigray, Amhara, Oromi, Southern Nations Nationalities and Peoples Region (NNPR), Harari, Dire Dawa, and Addis Ababa based on their geopolitical characteristic^38^. For simplicity of analysis, other independent variables were recorded.

Other community level variables such as community-level women’s education, community-level poverty, community-level media exposure, community-level modern contraceptive awareness were generated by aggregating the individual level variables data. Community-level variables were classified as “high” or “low” by using median value as cut-off point since the data was not distributed symmetrically. The community poverty level was classified as high when the proportion of reproductive women in the lowest wealth quintiles was greater than the median value, and low when the proportion was lower than the median value^39^. Similarly, community level of women’s education was classified as high when the proportion of women with at least primary level education was higher than the median cut-off value whereas considered as low when it was lower than the median value^40^. Moreover, community level media exposure was categorized as high media coverage when the proportion of women who had at least one media exposure, and low otherwise. Low-level media coverage was coded as “0” and otherwise “1”^41^.

### Data processing and analysis

Data extraction and cleaning, coding, labeling and analysis were performed by using Stata version 14 software (StataCorp, College Station, Texas 77,845 U.S.A which is available at http://www.stata.com). Weighting factor (v005) was used to decrease the effect of sampling bias that was raised because of unequal probability selection of strata and non-response.

Multi-level analysis was done after ascertain the eligibility of data by using Inter-cluster Correlation Coefficient (ICC) to consider the hierarchical nature of the DHS data. Bi-variable multi-level logistic regression analysis was done to compute the crude odd ratio at 95% confidence level and variables with p-value < 0.20 were candidate for multi-variable multi-level logistic regression analysis. Multivariable multi-level logistic regression analysis was employed after identified eligible variables with four well fitted serious of model. The four models includes null model (empty model; without independent variable); analyze only the unintended pregnancy through cluster variable to examined the random effect, Model I (examined the effect of only individual-level factors), model II (examined the effect of only community-level factors), and model III (examined the mixed effect of individual and community factors at the same time). Statistical association for fixed effect of variables on unintended pregnancy was declared at p-value < 0.05 with adjusted odd ratio (AOR) at 95% confidence interval.

The variations between clusters (random effect result) were estimated using three different methods such as Intra-Class Correlation (ICC), Median Odd Ratio (MOR) and Proportional change Variance (PCV). ICC was done for each four models and it shows the variation of community characteristics effect on unintended pregnancy of reproductive age women in rural areas. ICC was calculated by using the formula of ICC= [(V_A_)/ (V_E_+3.29)] × 100, where V_A_ represents the area/cluster-level variance, and V_E_ represents the individual-level error variance^42^. The higher ICC value helps to identify the most relevant community characteristics to realize the individual variation in unintended pregnancy.

MOR refers the odd ratio between the two randomly selected different areas with highest risk and the lowest risk and it was computed by using the formula of MOR = ${e^{0.95\sqrt {\operatorname{var} iance} }}$. The MOR value indicates the level of risk of an individual being resident to that area to have unintended pregnancy of reproductive women^43^. PCV was done for model I, II, III with respect the variance the model zero to portray the power of factors in the model to examine unintended pregnancy and PCV was computed by subtracting the variance of each model from the empty model by using the formula PCV= (Ve−Vmi)/Ve where Ve; variance of the empty model and Vmi; variance of consecutive model^44^. Model fitness for the three models was examined by using nested deviance.

The missing values were handling based on EDHS guideline. Missing value of current pregnancy intention status was excluded from the numerators and it was included in the denominator. If the outcome variable; current pregnancy intention status, contained the missing or “no response” or “don’t know” considered as undesired pregnancy. Explanatory variables that include missing value more than 5% excluded from further analysis since the EDHS survey is cross-sectional study.

### Patient and public involvement statement

Currently pregnant women were included in this study by furnishing valuable information. However, the study participant have never been participated in the study design, protocol, data collection tools, and disseminating this finding.

## Result

### Individual level characteristics of the rural pregnant women

A total of 974 reproductive aged women were included in this analysis. The overall mean age of the women was 27.9(SD ± 6.4) years and about three-fourth (75.2%) of them were between 20 and 34 years of age. More than half (59.6%) of the women didn’t attend formal education. All most all (96.15%) of the women were married and about 45% the women were Muslim followers. Besides, around 55.8% of women had work but; slightly higher than half (53.2%) of women had poor wealth status. Regarding to household head, majority (87.87%) of the household head were male. Moreover, nearly half (48.3%) of women had 4–6 family size and nearly, two-third (65.9%) the women had desire to have more children (Table 1).

**Table 1:** Individual level characteristics of pregnant women in EDHS 2016.

Variable	Frequency (weighted)	Percentage (weighted)
Age		
15–19	78	8.0
20–34	732	75.2
35–49	164	16.8
Maternal education		
No formal education	580	59.6
Primary	347	35.6
Secondary and above	47	4.8
Religion		
Orthodox	292	30.0
Muslim	442	45.3
Protestant	208	21.4
Catholic and others	32	3.3
Marital status		
Married	937	96.15
Not married	37	3.85
Maternal occupation		
Working	544	55.84
Not working	430	44.16
House wealth index		
Poor	518	53.15
Middle	209	21.50
Rich	247	25.35
Media exposure		
Yes	277	71.58
No	697	28.42
Household family size		
1 to 3	259	26.59
4 to 6	471	48.34
7 and above	244	25.07
Sex of household head		
Male	856	87.87
Female	118	12.13
Parity		
Primiparous	299	30.72
Multiparous	418	42.88
Grand multiparous	257	26.40
Ever terminated pregnancy		
Yes	878	90.16
No	96	9.84
Intention to contraceptives		
Intend to use	689	70.75
Does not intend to use	285	29.25
Unmet need for contraception		
Not unmet need	666	68.33
Unmet need	308	31.67
Awareness on modern contraceptive method		
Knows no method	28	2.87
Knows modern method	946	97.13
Desire for another child		
Yes	642	65.94
No	332	34.06
Knowledge of ovulation cycle		
Know ovulation period	787	80.81
Don’t know ovulation period	187	19.19

*Yes; if the women want to have another child within 2 years or after 2 years.

*No; if the women do not want any more children.

### Community level characteristics

All most all (92.67%) of women were from larger central region and most (62.37%) of the women perceived that they had big problem to get medical care at the health facility. Most (61%) of the community women educational status was low and more than half (53%) of community women had media exposure. Moreover, nearly, two-third (66.17%) of the community women wealth status were poor (Table 2).

**Table 2:** Community level characteristics of pregnant women in Ethiopia, EDHS 2016.

Variable	Frequency (weighted)	Percentage (weighted)
Distance to the health facility		
Big problem	608	62.37
Not big problem	366	37.63
Region		
Small peripheral	71	7.33
Larger central	903	92.67
Community media exposure		
Yes	516	52.98
No	458	47.02
Community women educational level		
Higher women education	380	39.01
Low women education	594	60.99
Community awareness on modern contraceptive method		
High	70	7.18
Low	904	92.82
Community wealth status		
Rich	329	33.83
Poor	645	66.17

### The prevalence of unintended pregnancy

**Fig. 2:**
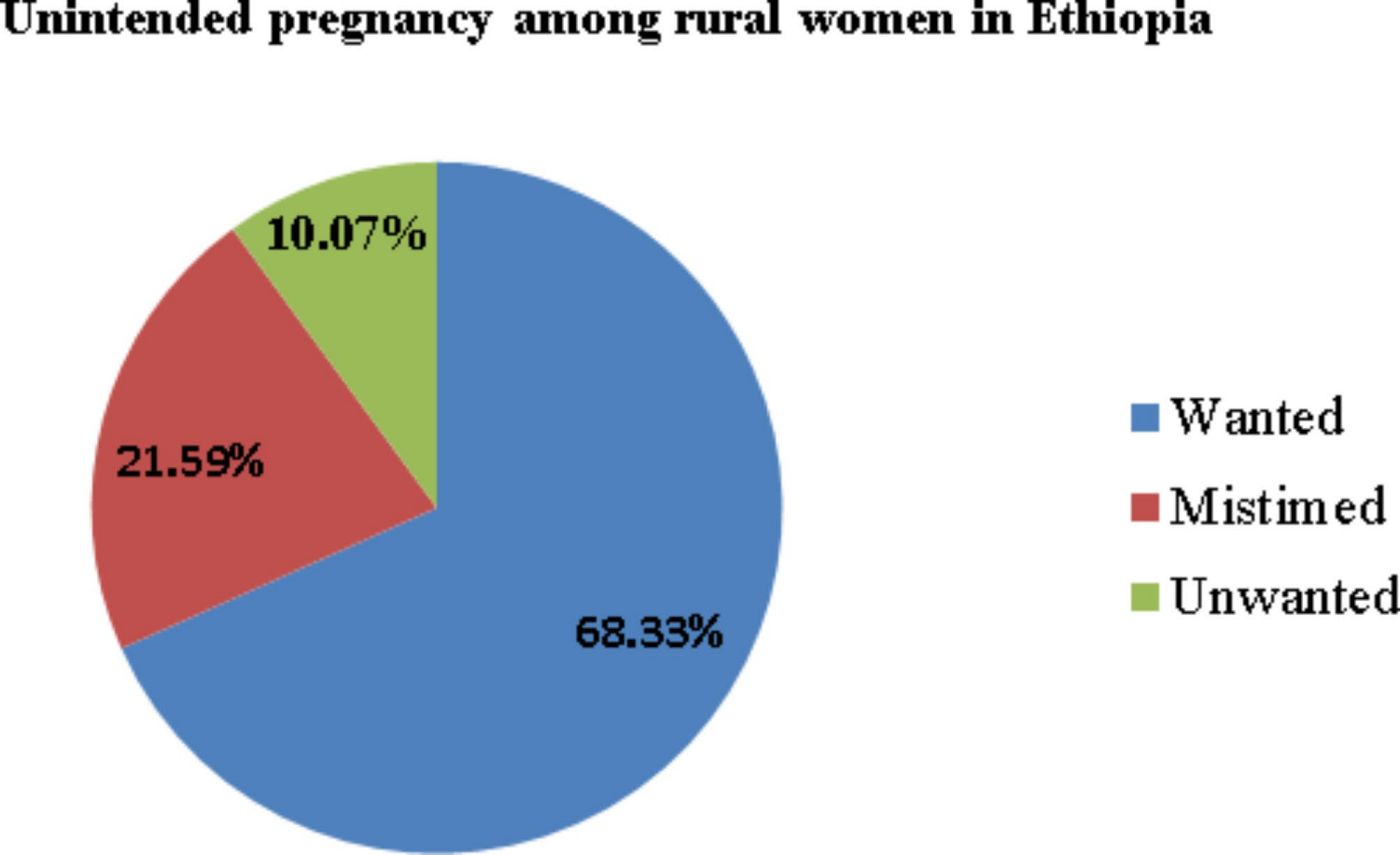
Prevalence of pregnancy intention among pregnant rural women in Ethiopia, EDHS 2016.

In this study, the overall prevalence of unintended pregnancy among rural reproductive-age women was 31.66%( 308), 95% CI 28.81%, 34.66%) comprises 21.59% mistimed and 10.07% of unwanted (Fig. 2).

### Random effect and model fitness

The Intra-class correction (ICC) of null (empty) model was 57.52% which indicates that the overall variability of unintended pregnancy can be attributed to cluster variability. The median odd ratio (MOR) of unintended pregnancy in the empty model was 6.11. This portrayed that if an individual randomly selected from two different clusters and then those women from clusters having high unintended pregnancy had 6.11 times higher odds to have unintended pregnancy as compared to women from the lower unintended pregnancy clusters. Besides, the proportional change variance (PCV) increase from 26.99% in model I to 38.29% in model III, this shows the final model (model III) best explained the unintended pregnancy variability. The model fitness was checked by using deviance (-2LLR) and model III was the best fitted model since it had the lowest deviance value (Table 3).

**Table 3:** Model comparison and random effect analysis result of unintended pregnancy among rural women in Ethiopia, EDHS 2016.

Random effect	Null model	Model I	Model II	Model III
Variance	3.63	2.65	2.99	2.24
ICC (%)	57.52%	44.61%	47.62%	40.54%
MOR	6.11	4.69	5.17	4.14
Explained variance (PCV)	Ref	26.99%	17.63%	38.29%
Model fitness				
Deviance (-2logliklihood)	995.88	1100.98	1067.26	964.38

### Individual and community-level factors associated with unintended pregnancy (fixed-effects)

Individual-level and community level factors were adjusted in the final model (model III). Individual-level factors such as maternal occupation, maternal media exposure, maternal wealth status, household family size, parity, and contraception intention of the women were statistically associated with unintended pregnancy of rural women. Among community-level factors, region and community level wealth were statistically associated with unintended pregnancy.

In the fixed effect model, in the bi-variable multilevel modeling, all individual-level and community-level factors which had *p* < 0.2 were exported to multi-variable multilevel logistic regression analysis. Those women who never had occupation were 67% lower to had unintended pregnancy than women who had occupation (AOR: 0.33, 95%CI 0.21, 0.52). Women from poor household wealth were 2.4 times high risk to exhibited unintended pregnancy when compared to women from rich household wealth (AOR: 2.4, 95%CI 1.24, 4.56). Likewise, women who never had media exposure were 2.67 times high risk to experienced unintended pregnancy as compared to the counterparts (AOR: 2.67, 95% CI 1.48, 4.83). Additionally, women from household family size of one to three members were 56% lower to had unintended pregnancy than women from household family size of seven and above members (AOR: 0.44, 95% CI 0.2, 0.96). Besides, the likelihood of unintended pregnancy was 59% lower among primiparous women compared to grand multiparous women (AOR: 0.41, 95% CI 0.17, 0.99).

Similarly, the odds of unintended pregnancy were 76% lower among women who never had intention to use contraceptive when compared to women who had intention to use contraceptive (AOR: 0.24, 95%CI 0.14, 0.41). Moreover, those women who lived in larger central region were 4.2 times high risk for unintended pregnancy as compared to women who reside in small periphery region (AOR: 4.2, 95% CI 1.19, 14.62). Furthermore, the odds of unintended pregnancy were 4.3 times higher among women from poor community level wealth as compared their counterparts (AOR: 4.3, 95% CI 1.85, 10.22) (Table 4).

**Table 4:** Multi-variable analyses for factors affecting unintended pregnancy among rural reproductive age women in Ethiopia (*n* = 974).

Variables	Pregnancy intention		Model I AOR (95% CI)	Model II AOR(95% CI)	Model III AOR(95% CI)	
	Unintended *n* (%)	Wanted *n* (%)				
Age						
15–19	15	62	1		1	
20–34	228	505	0.8(0.32, 2.01)		0.9(0.36, 2.26)	
35–49	65	99	1.53(0.49, 4.82)		1.44(0.45, 4.61)	
Maternal educational status						
No formal education	188	392	1.05(0.36,3.08)		1.1(0.35, 3.17)	
Primary	107	240	1.19(0.41, 3.41)		1.09(0.38, 3.13)	
Secondary and above	13	34	1		1	
Occupational status						
Not working	139	405	0.34(0.22 0 0.54)		0.33(0.21, 0 0.52)**	
Working	169	261	1		1	
Wealth status						
Poor	163	355	1.55(0.84, 2.88)		2.4(1.24, 4.56)**	
Middle	62	147	0.81(0.42,1.56)		0.83(0.44, 1.59)	
Rich	83	164	1		1	
Media exposure						
Yes	228	469	1		1	
No	80	197	2.45(1.40, 4.26)		2.67(1.48, 4.83)**	
House hold size						
1 to 3	53	206	0.55(0.26,1.19)		0.44(0.20, 0.96)**	
4 to 6	163	308	0.83(0.45,1.56)		0.68(0.37, 1.28)	
7 and above	92	152	1			
Parity						
Primiparous	64	235	0.4(0.17, 0.97)			0.41(0.17, 0.99)**
Multiparous	138	280	0.8(0.40 1.58)			0.82(0.41, 1.61)
Grand multiparous	106	152	1			1
Intention to contraceptives						
Intend to use	256	434	1			1
Does not intend to use	52	232	0.18(0.11, 0 0.31)			0.24(0.14, 0 0.41)**
Region						
Small peripheral	5	71		1		1
Large central	303	595		6.5(2.09, 20.29)		4.2(1.19, 14.62)**
Distance to the health facility						
Big problem	216	392		1.8(1.13, 2.75)		1.64(0.98, 2.74)
Not big problem	92	274		1		1
Community level educational status						
Higher	100	280		1		
Low	208	386		0.9(0.47, 1.63)		1.19(0.56, 2.56)
Community level wealth status						
Poor	230	415		2.6(1.29, 5.11)		4.3(1.85, 10.22)**
Rich	78	251		1		1
Community level media exposure						
Ever had media exposure	166	350		1		1
Never had media exposure	142	316		0.9(0.49 1.64)		0.57(0.27 ,1.22)

** Statistically significant.

*AOR* adjusted odd ratio, * 1* reference.

## Discussion

The current study aimed to assess the prevalence and associated factors of unintended pregnancy among rural women in Ethiopia using EDHS 2016 data through multilevel analysis. The overall prevalence of unintended pregnancy was 31.66% which is comparable with study conducted in national report of EDHS 2011^33^, Shashmanie, Ethiopia^45^, Egypt^46^ and Sab Saharan Africa^47^. This study finding is less than the study conducted in Gellan^48^, South Africa^49^, Botswana^50^, Arsi, Ethiopia^29^, Ghana^22^, Addis Ababa^51^ and Debere Markos^52^. The discrepancy might be due to study population and study period variation, study area difference, methodological variation, difference in maternal health quality, coverage and accessibility including family planning methods, and socio-cultural. This study includes merely the rural women whereas study done in Gellan, Arsi, Debere Markos and Ghana were community based that include both rural and urban that may increase magnitude of unintended pregnancy on those study areas. On the other hand, study report in Addis Ababa revealed that most study participant that had unintended pregnancy were women in schools whereas the current study majority of the participants were not attend formal education. Thus, women in schools are the most fire age groups, influenced by peers’ pressure and have access to watch pornography that induces sexual desire for those adolescent girls to have sex and may not have awareness about family planning that leads to unintended pregnancy and abortion. Besides, study conducted in Debere Markos, Ethiopia showed that the study participants had risky behavior such as substance abuser, alcohol drunker and khat user’s practices risky sexual behavior that attribute for unintended pregnancy whereas this study participants are rural women who had less to zero risky behavior for addiction. Moreover, by fearing social discrimination, the cultural taboo and intention of husband to have more children, rural women have a tendency of saying the pregnancy was wanted after the pregnancy already occurred even if they didn’t want really.

However; this finding is higher than study done in low and middle income countries^53^, Ghana^9^, Gambia^54^, Ethiopia national survey 2013^33^, Gondar town^27^, Bangladesh^15^, Europe^55^, and Cambodia^21^. The possible reason for the difference between in this study and Europe might be there is a governmental policy in Europe to empower women to have autonomy and access to contraception. Women who have more autonomy are more likely to be aware about contraceptive methods, which increases the probability that women will use contraceptives and this may leads to reduction of the risk of unintended pregnancy^56,57^. Likewise, European women have better chance of inter-spouse communication regarding family planning^58^ in contrast, rural women in Ethiopia may have less chance of open communication with her husband about the family planning. Additionally, Ethiopian couple’s women have low decision-making autonomy on contraception and their fertility that may contribute for unintended pregnancy^59,60^. Study done in Bangladesh revealed that all study participates were married whereas the current study were both married and unmarried. Indeed, married women are less likely to have unintended pregnancy than unmarried women. In addition, study done in rural Ghana showed that there was inadequate reproductive health rights, low empowerment of women and poor access to family planning services^9^. The uptake of contraceptive in rural women in Ethiopia is 44.6%^61^ whereas in rural Ghanaian’s women is 27.8%^62^. Additionally, women’s decision-making power regarding family planning in Ethiopia was 57% whereas majority(89%) of Ghanaian’s women were disempowered and about 72% of them do not decided on their own healthcare alone^63^.

Furthermore, Southwest Ethiopia study was institutional based that may underestimates of the unintended pregnancy in contrast this study was community based that may show real figures of unintended pregnancy.

In multivariable multilevel logistic regression analysis household size, media exposure, maternal occupational status, wealth status, parity, contraception intention, region and community level wealth.

Women from household family size of one to three had lower odds of unintended pregnancy compared to women from household family size of seven and above. This study finding is similar with study done in Six Asian countries^64^, Ethiopia^27,31^, Sudan^65^ and Nigeria^32^. The possible justification for the variation could be women from smaller household family size assumed to have adequate resources and this makes the women to access contraceptive services and family planning education more easily. Additionally, women in smaller household family size are deemed to be educated and have autonomy in decision-making to limit the spacing and the number of their children^66^. Thus, women are less likely to get unintended pregnancy since they are more likely to use family planning. Moreover, women in smaller household family size may have more time to access information regarding family planning service as compare to women with larger household family size since they spent hard time in care of their families and children^38^.

Women who didn’t have media exposure were more likely to have unintended pregnancy. This finding is in line with study done in Ethiopia^67,68^, Nepal^69^ and Pakistan^70^. The potential reason may be women who don’t have media exposure may not got information regarding to contraceptive methods and importance of maternal health service utilization. This implies that concerned body has to give attention maternal media exposure in those marginalized rural community.

Women who are not currently working have lower odds of unintended pregnancy compared to women who are currently working. This result is congruent with a study done in Ethiopia^71,72^. However this finding is contradictory to study done in Ethiopia^73^ and Cambodia^21^. The potential explanation for the variation might be women who don’t have work could have low level of social contact and self-confidence to have relationship, and may be free from the influence of work that induces to practice unplanned sex that in turn leads to unintended pregnancy^71^. Additionally, women who don’t have work have low household income and they more likely to use family planning since they may perceived that they can’t afford to welcome another child rather they may prefer to delay family expansion. Moreover, currently in Ethiopia, women who don’t have work are eligible for community based health insurance and health insurance coverage is high in rural dwellers^74^, and family planning service are exempted service in public health facilities^75^. This may enable the women to use family planning service, which in turn decrease the likelihoods of unintended pregnancy. Furthermore, women who don’t have work may have low work-related stress as result they may got chance to get reproductive health education and unmarried women might be less likely to have partner or be in relationship, and may be under familial or community based scrutiny about their reproductive behavior that may limit their sexual activity that in turn decrease likelihood of unintended pregnancy.

This study also highlighted that primiparous women were less likely to have unintended pregnancies when compared to grand multiparous women. This finding is consistent with study done in Debre Markos^52^, Ethiopia^76^, Harar, Ethiopia^77^. A possible explanation might be that primiparous women exhibited their first pregnancy as result they may accept the pregnancy; hence they may report the pregnancy as wanted whereas grand multiparous women may deemed that they reach tsheir desire number of children; afterwards of any pregnancy may reported as unplanned^78^. Additionally, now a days, first-time mother are more often younger and attended formal education, might have mobile phone and access to use internet; thus women are more likely to exposed to family planning messages^79,80^. Similarly, primi women may want to delay pregnancy by using family planning so as to decrease overburden; due to pregnancy and childbearing, since they may want to contribute to family’s livelihood^81^ and to pursue their education. This finding suggested that community health workers should provide counselling service to grand multiparous women regarding family planning during postpartum period visit to health facility or during home to home visit.

Poor women have higher odds of unintended pregnancy compared to rich women and this finding is consistent with other study done in Nigeria^32^, Venator^82^, India^83^ and South Asia^64^. The possible reason may be poor women may not have opportunity to attained formal education, less likely to use contraceptive and high risk for unprotected sex that leads to unintended pregnancy. This implies that policy maker and planners has to design strategies to address those low income level rural women.

Women who didn’t have intention to use contraceptive were less likely to have unintended pregnancy than women who had intention to use contraceptive. This result is inconsistent with study in South Asian countries^64^. The plausible justification for the discrepancy might be women who didn’t have intention to use contraceptive would perceive themselves as low risk to get pregnancy and they may also gave socially desirable response for already occurred pregnancy by responding didn’t have intention to use contraception. This finding is contradictory to study done in Ethiopia^84^. Moreover, women residing in large central region were more likely to have unintended pregnancy as compared to women residing in small periphery regions. This finding is similar with other studies done in Ethiopia^31^, Kenya^20^ and Ghana^22^. The variation might be due to population size in small peripheral region is lower as compared to larger central region, thus women’s pregnancy may be recommended and acceptable in small peripheral region whereas women in the large central region women may be busy due to their intention to improve their financial status and majority of the women’s pregnancies tends to be unintended^38^. Furthermore, community wealth status was another community level factor associated with unintended pregnancy. Women from poor community-level had higher odds of unintended pregnancy compared to counterparts and it is consistent with other similar study^64^. The discrepancy may be women from poor community-level may not have access to education and media exposure as result women may not have chance to obtain information about family planning. In addition, lack of transportation cost to utilize maternal health service may contribute unintended pregnancy.

### Strength and limitation of this study

The present study had strength because it used large nationally representative data. This study used the appropriate multilevel statistical approach to account the hierarchical nature of the data. Since this study used nationally representative data, it has high chance of providing insights for policy-makers and program designers to set the most potential intervention strategies at all levels(nationally and regional level). This study might have the possible limitation of recall bias since this study used the EDH survey data that was heavily relay on the respondent’s self-report. Moreover, this study was unable to ascertain the temporal relationship between unintended pregnancy rather it shows the association between unintended pregnancies and factors.

### Conclusion and recommendations

The present study prevalence of unintended pregnancy in rural women was relatively high. From multivariable multilevel logistic regression analysis, individual level variable such as maternal occupation status, household size, media exposure, parity, wealth status and contraception, and community level variables such as region and community level wealth were statistically associated with unintended pregnancy. Hence, priority attention has to be given to increase accessibility of media and set strategies to improve women financial capacity to those high vulnerable groups (unintended pregnancy) and enhancing availability maternal health service to decrease adverse outcome of unintended pregnancy in rural areas.

## Data Availability

The data used for the present study will be accessible with rational request from responsible corresponding author and every one can access the data set online from www.measuredhs.com.

## Abbreviations

AOR: Adjusted odds ratio
CI: Confidence interval
DHS: Demographic and health survey
EAs: Enumeration areas
EDHS: Ethiopian demographic and health survey
ICC: Intra class correlation
MOR: Median odd ratio
PCV: Proportional change in variance
PHC: Population and housing census
SNNPR: Southern nations nationalities and peoples region
